# P-1867. Under-Recognized Medication Loss with the Administration of Small-Volume Intermittent Infusions

**DOI:** 10.1093/ofid/ofae631.2028

**Published:** 2025-01-29

**Authors:** Aamir S Dave, Patricia Saunders-Hao, Sumeet Jain, Michele Graci, Evelyn Luo

**Affiliations:** SUNY Downstate, Hicksville, New York; North Shore University Hospital, Manhasset, New York; North Shore University Hospital, Manhasset, New York; North Shore University Hospital (Manhasset, NY), Manhasset, New York; North Shore University Hospital, Manhasset, New York

## Abstract

**Background:**

Most antimicrobials administered via intermittent intravenous (IV) infusion are diluted in 50 to 100 mL of diluent. The primary infusion set for a smart pump can hold 25 mL of volume in its tubing, which can contribute up to a 50% drug loss if residual volume is present after administration is complete. For antimicrobials, this may lead to significant underdosing, contributing to poor treatment outcomes and antimicrobial resistance. Many organizations lack departmental policies and procedures (P&P) to ensure complete administration of small-volume intermittent infusions (SVIIs). Previous studies propose several recommendations, such as adding carrier fluids and flushing the line, but there is no easy fix to this common issue.

**Methods:**

Our aim was to implement new guidance in 2 pilot patient units (an ICU and non-ICU) to address the administration of SVIIs and evaluate its effect on medication administration. This was an observational quality improvement initiative assessing the new guidance established for the administration of SVIIs to the current practices at North Shore University Hospital. The new guidance consisted of nursing education to reinforce proper administration techniques and to adjust infusion volumes for overfill. This study was IRB exempt. The primary outcome of this study was the incidence of residual drug volume in IV line before the air-detector, IV bag, or IV vial. This was done through direct observation and data was collected through a survey. Chi-square tests were used to analyze the primary outcomes.

**Results:**

In total, 203 IV administrations were observed, 86% were antimicrobials. There were 124 IV administrations observed before policy guidance initiation, and 79 after initiation. There was a significant reduction in the incidence of fluid remaining in the IV line before the air-detector (85% vs 27%; p-value < 0.001), the IV bag (59% vs. 7.6%; p-value < 0.001), and in the IV vial (47% vs. 24%; p-value < 0.001).

**Conclusion:**

The interventions significantly decreased the incidence of fluid remaining in the IV line, presumably decreasing medication loss. Since most medications were antimicrobials, this may improve clinical outcomes. This will be used to update P&P to standardize IV administration practices and improve patient care.

**Disclosures:**

All Authors: No reported disclosures

